# Development of an exosome-related and immune microenvironment prognostic signature in colon adenocarcinoma

**DOI:** 10.3389/fgene.2022.995644

**Published:** 2022-09-13

**Authors:** Guoliang Cui, Can Wang, Jinhui Liu, Kinyu Shon, Renjun Gu, Cheng Chang, Lang Ren, Fei Wei, Zhiguang Sun

**Affiliations:** ^1^ Department of Gastroenterology, The Second Affiliated Hospital of Nanjing University of Chinese Medicine, Nanjing, Jiangsu, China; ^2^ Department of Colorectal Surgery, Affiliated Hospital of Nanjing University of Chinese Medicine, Jiangsu Province Hospital of Chinese Medicine, Nanjing, Jiangsu, China; ^3^ Department of Gynecology, The First Affiliated Hospital of Nanjing Medical University, Nanjing, Jiangsu, China; ^4^ School of Traditional Chinese Medicine and School of Integrated Chinese and Western Medicine, Nanjing University of Chinese Medicine, Nanjing, Jiangsu, China; ^5^ Department of Physiology, School of Medicine and Holistic Integrative Medicine, Nanjing University of Chinese Medicine, Nanjing, Jiangsu, China

**Keywords:** colon adenocarcinoma, prognosis, exosome-related gene, tumor immune microenvironment, immunotherapy

## Abstract

**Background:** The correlation between exosomes and the tumor immune microenvironment has been proved to affect tumorigenesis and progression of colon adenocarcinoma (COAD). However, it remained unclear whether exosomes had an impact on the prognostic indications of COAD patients.

**Methods:** Expression of exosome-related genes (ERGs) and clinical data were downloaded from The Cancer Genome Atlas (TCGA) and Gene Expression Omnibus (GEO) database. The ERGs associated with prognosis were identified and exosome-related prognostic signature was constructed. Patients in two risk groups were classified according to the risk score calculation formula: Risk score = 1.0132 * CCKBR + 0.2416 * HOXC6 + 0.7618 * POU4F1. The expression of three ERGs was investigated by qRT-PCR. After that, we developed a nomogram predicting the likelihood of survival and verified its predictive efficiency. The differences of tumor immune microenvironment, immune cell infiltration, immune checkpoint and sensitivity to drugs in two risk groups were analyzed.

**Results:** A prognostic signature was established based on the three ERGs (CCKBR, HOXC6, and POU4F1) and patients with different risk group were distinguished. Survival analysis revealed the negative associated of risk score and prognosis, ROC curve analyses showed the accuracy of this signature. Three ERGs expression was investigated by qRT-PCR in three colorectal cancer cell lines. Moreover, risk score was positively correlated with tumor mutational burden (TMB), immune activities, microsatellite instability level, the expression of immune checkpoint genes. Meanwhile, the expression level of three ERGs and the risk score were markedly related with the sensitive response to chemotherapy.

**Conclusion:** The novel signature composed of three ERGs with precise predictive capabilities can be used to predict prognosis and provide a promising therapeutic target for improving the efficacy of immunotherapy.

## Introduction

COAD is the ordinary histological subtype of Colorectal cancer (CRC) (Siegel et al., 2021). Among the top four malignant tumors in the world, nearly 900 000 COAD patients die annually (Dekker et al., 2019). In addition, it is estimated that the incidence of colon cancer in patients aged 20–34 will increase by 90.0% in 2030 (Siegel et al., 2021). More and more attention has been paid to the early diagnosis and prevention of COAD (Benson et al., 2018). Biomarkers are rapidly being discovered through the study of the transformation process of COAD. Previous studies have revealed that M2 macrophage-derived exosomes (MDE) and cancer-associated fibroblasts related exosomes regulated the migration and invasion of CRC cells (Hu et al., 2019; Lan et al., 2019), circulating exosome microRNAs can also reflect pathological changes in COAD patients (Ogata-Kawata et al., 2014). However, large-scale clinical trials are required to verify these tumor biomarkers before they can be used in clinic. Overall, the pathogenesis of COAD is complex, and more researches are needed to further explore the signature of exosomes associated with COAD to improve the accuracy of early diagnosis and prediction.

As a type of extracellular vesicles (EV), exosomes are 30–150 nm-sized vesicles, surrounded by lipid bilayer and containing a variety of biomolecules (Zhang et al., 2015). In the past few years, the important role of exosomes in tumor progression, initiation, metastasis and immunity has been deeply discussed (Möller and Lobb, 2020). First of all, exosomes not only regulate gene expression level of cancer cells (Farooqi et al., 2018), but also directly affect cell polarity and directional cell movement, so as to establish a good ecological niche of cancer cells before metastasis in tumor microenvironment (TME) (Rana et al., 2013). Exosomes are important mediators in the translation of inflammation related colorectal cancer (Zhang et al., 2018), and the previous studies suggested that cancer cell-derived exosomes could regulate tumor immune response by indirectly or directly activating T-cell function (Zhang and Yu, 2019). In addition, exosomes are associated with immune escape that can be driven by tumor associated macrophages and neutrophils (Kim and Bae, 2016). Interaction between exosome-carried programmed death ligand 1 (PD-L1) and T-cells that produce programmed death 1 (PD-1), significantly reduce the response to immune checkpoint blocking drugs (Lee et al., 2022). Consequently, exosomes have emerged as one of the key mechanisms regulating the immunotherapy of malignant tumors and it is crucial to investigate exosome-related prognostic signature in COAD patients.

In this study, we integrated ERGs expression profiles and clinical information of COAD patients to construct a novel signature based on 3 key ERGs for prognosis prediction, references for clinical chemotherapy and immunotherapy.

## Materials and methods

### Data acquisition

The gene expression profiles and the clinical information of the COAD patients were retrieved from TCGA database (Wang et al., 2016) and the matching information from the GSE39582 dataset was availed from GEO database (Barrett et al., 2013). Therefore, 514 samples which included 41 normal samples from TCGA, and 585 samples which contained 566 cancer samples and 19 normal samples (557 samples had prognostic information) from the GEO were introduced for further study.

### Differentially expressed exosome-related genes and functional annotation

The “limma” software package in R software was used to analyze the differentially expressed genes (DEGs) of 121 ERGs which were acquired from ExoBCD database (https://exobcd.liumwei.org/) (Qiu et al., 2021). The genes with threshold of |log2FC| ≥ 1 and FDR ≤ 0.05 were treated as the DEGs. Univariate Cox regression and Kaplan-Meier (KM) survival analysis were used to identify the prognostic DEGs with *p* less than 0.05. ClusterProfiler package of R software for Gene Ontology (GO) analysis and Kyoto Encyclopedia of Genes and Genomes (KEGG) pathway analysis were utilized to identify the biological functions of the prognostic DEGs.

### Establishment and validation of an exosome-related prognostic signature

After removing the COAD samples from TCGA database with unknown survival time, and unknown survival status, a total of 417 patients were enrolled by integrating the transcriptome and clinical data in this study. At a ratio of 1:1, half of the samples randomly obtain from the entire set (*n* = 417) containing all COAD samples were assigned to the training set (*n* = 209) and the other half to the testing set (*n* = 208). No significant difference was observed among the entire set, training set and testing set for the clinical-pathological factors (Supplementary Table S1). After the prognostic signature was constructed in training set, we performed model validation on the test set, the entire set, and the GEO set to improve accuracy. Univariate cox regression analysis was utilized to identify DEGs associated with overall survival (OS). We evaluated the performance of LASSO regression using 10-fold cross-validation approach in our analysis, *p*-value of 0.05, as well as running 1,000 times. “Glmne” package was used for LASSO regression analysis to further identify key ERGs. The coefficient of each gene analysis was extracted for the establishment of the prognostic signature by multivariate Cox regression. After that, we calculated the risk score with the formula: Risk score=∑coef∗Exp(genes) . The median score served as a demarcation line to help us divide COAD patients into high-risk and low-risk groups. The Kaplan-Meier (K-M) analysis was performed with the “survival” package to investigate the correlation of risk score and OS. ROC analysis was performed to reveal the sensitivity and specificity of exosome-related signature and principal component analysis (PCA) was used for dimensionality reduction.

### Cell culture

The colorectal cancer cell lines (Caco-2, HT-29, and HCT116) were purchased from China Center for Type Culture Collection (CCTCC). The normal colorectal cell line (FHC) was obtained from Cell Bank of Type Culture Collection of the Chinese Academy of Sciences (Shanghai, China). Caco-2, HT-29, HCT116, and FHC cells were cultured in McCoy’s 5A, RPMI-1640, DMEM respectively (Gibco, China) containing 10% fetal bovine serum (Gibco, China) in a humidified incubator at 37°C and 5% CO_2_.

### qRT-PCR

TRIZOL reagent (Thermo Fisher Scientific, United States) was used to isolate total RNA from cell lines and complementary DNA (cDNA) was synthesized using Revert Aid First Strand cDNA Synthesis kit (Vazyme, China). qRT-PCR was performed using ChamQ Universal SYBR qPCR Master Mix (Vazyme, China) and β-actin was chosen as the internal referenece. The relative expression of the target gene was estimated using the 2^−ΔΔCT^ method. The primer sequences are listed in Supplementary Table S2.

### Construction of a predictive nomogram

The Nomogram was generated by R software with the “rms” package. The “rms” R package, risk scores, age, gender, and tumor stage were used to create a nomogram for different years OS (Iasonos et al., 2008). Calibration curves were conducted to assess the predictive value of the nomogram, and the closer the 45° line is, the better the prediction will be.

### Evaluation of tumor microenvironment

Tumor-infiltrating immune cells (TIICs) was estimated in a number of databases including TIMER, CIBERSORT, CIBERSORT-ABS, QUANTISEQ, MCPCOUNTER, XCELL, and EPIC. We download the profile of infiltration estimation from the TIMER 2.0 database to explore the relationship of risk score and infiltration of TIICs. The level of immune score and stromal score can be utilize to evaluate TME and its correlation with risk score was also revealed by ESTIMATE algorithm (Yoshihara et al., 2013). Single-sample gene-set enrichment analysis (ssGSEA) was utilized to quantify the difference of immune activity including tumors immune related cells and immune-related functions. One-class logistic regression (OCLR) machine-learning algorithm was used to quantify the stemness of tumor samples by calculating cancer stem cell indices Malta et al. (2018).

### Analysis of immune states

Tumor Immune Dysfunction and Exclusion (TIDE) score is based on the analysis of T-cell dysfunction in genes characterized by high levels of cytotoxic T-cell infiltration and T-cell rejection in immunosuppression to predict tumor immune evasion potential and tumor response to immune checkpoint inhibitors (ICIs). The TIDE score consists of two parts: The dysfunction score and the exclusion score (Jiang et al., 2018). Immunophenoscore (IPS) refers to regulation the immunogenicity, and is calculated impartially with machine learning methods (Charoentong et al., 2017). The potential responses of immune checkpoint inhibitors were compared by calculating IPS fractions based on gene expression levels of immune checkpoint genes through The Cancer Immunome Atlas (TCIA). Information of mutations, microsatellite instability (MSI), and tumor stem cells in each COAD patient was obtained from TCGA, the “maftools” packages was used to generate the waterfall diagram to show the mutation frequency of the top 10 mutated genes, the relationship between risk score and tumor mutational burden (TMB), immunogenicity was assessed.

### Drug sensitivity analysis

Through Genomics of Drug Sensitivity in Cancer (GDSC), boxplots show the response of patients in different risk groups to chemotherapy and small molecule compound (Yang et al., 2013). Half-maximal inhibitory concentrations (IC50) representing the drug response were estimated (Sebaugh, 2011). In this study, we referenced the NCI-60 database and the differences of drug sensitivity was explored. So far, the NCI-60 database is the most frequented database in relation to cancer drug testing and is accessible in CellMiner database (Hurwitz et al., 2016).

### Statistical analysis

All analyses were performed in R software (Yu et al., 2012). The continuous variables in normal distribution are analyzed by Student’s *t*-test, which is presented as mean ± standard deviation, and the continuous variables in abnormal distribution are presented as median (range). A *p*-value less than 0.05 was considered as statistical significance.

## Results

### Identification of differentially expression of exosome-related genes

**FIGURE 1:**
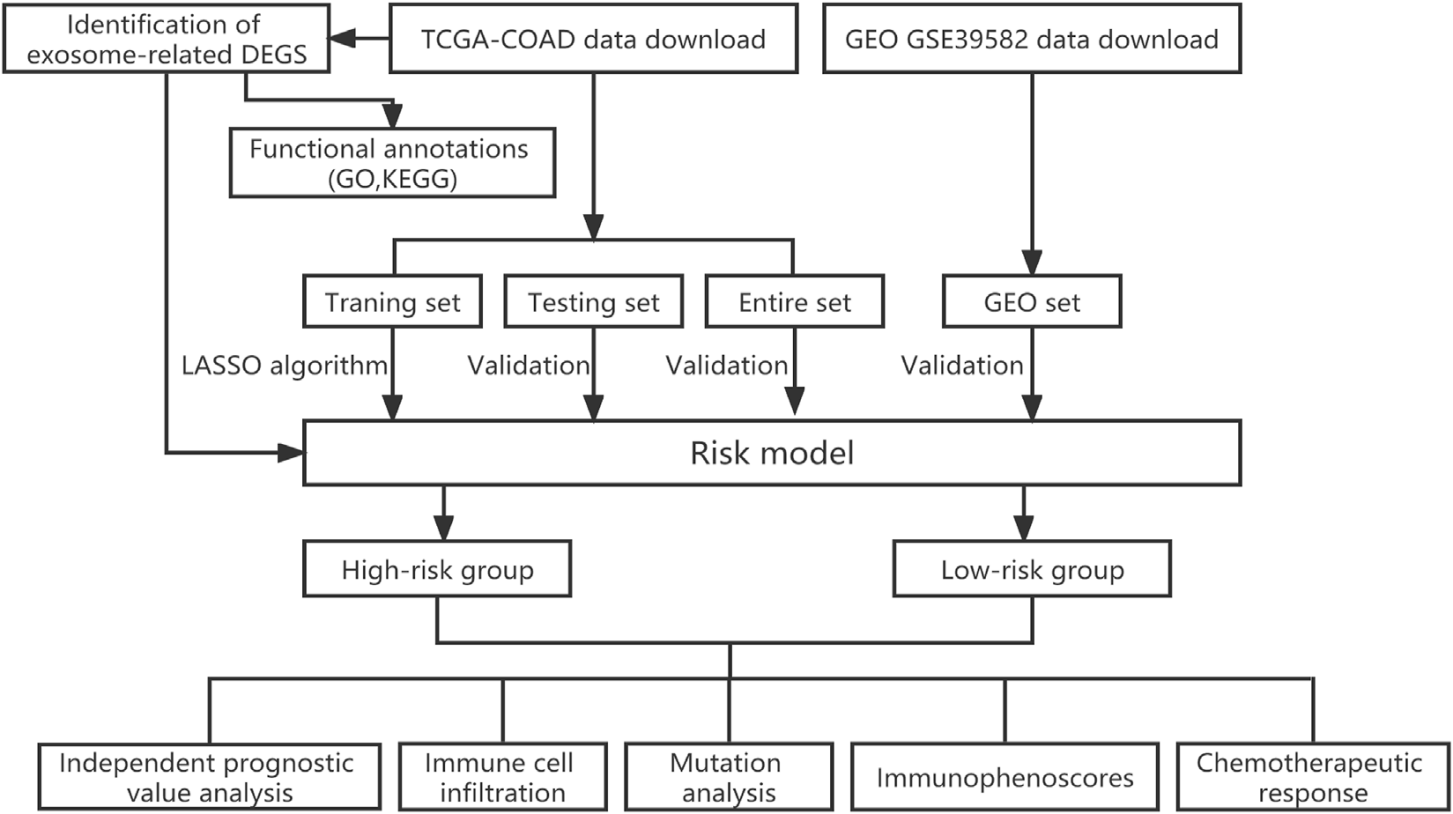
Flow chart of this study.

In order to better understand the methods and results of this study, a brief workflow was drawn (Figure 1). To screen the DEGs, 121 ERGs expression was investigated between the normal and tumor samples and the expression profiles of 30 differentially expressed ERGs were visualized in the heatmap, including 13 genes with upregulated and 17 genes with downregulated (Supplementary Figure S1A). Then, we performed enrichment analysis on exosome-related gene sets by GO function analysis. The biological process (BP) of was mainly involved in the cellular response to fibroblast growth factor, cellular response to xenobiotic stimulus, intrinsic apoptotic signaling pathway and regulation of DNA damage checkpoint. Cellular component (CC) was mainly concentrated in the excitatory synapse, and Molecular function (MF) was mainly related to sulfur compound binding, ubiquitin-protein ligase binding, heparin-binding and glycosaminoglycan binding (Supplementary Figure S1B). The results of KEGG analysis showed that ERGs were mainly enriched in the calcium signaling pathway, platinum drug resistance, inflammatory mediator regulation of TRP channels (Supplementary Figure S1C).

### Construction of exosome-related prognosis signature in training set

**FIGURE 2:**
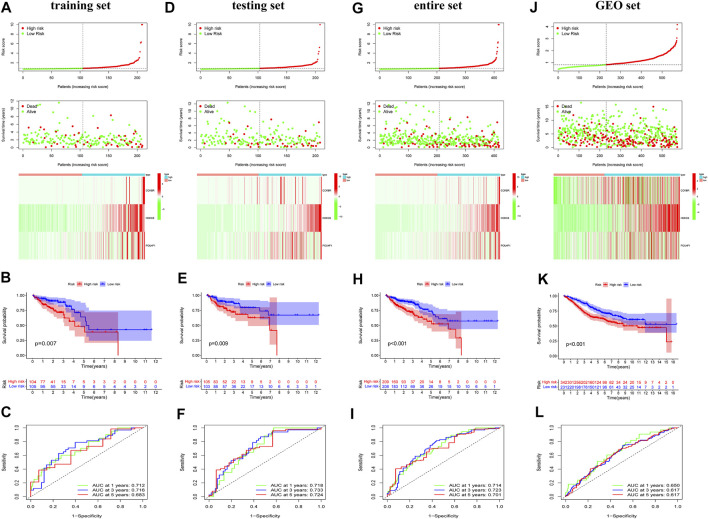
Identification and validation of the exosome-related genes signature. Risk scores for all patients in each cohort are listed in ascending order, with the median as the threshold, COAD patients were divided in low-risk (green) and high risk (red). The ranked dot plot illustrated the distribution of survival status and the heatmap showed the expression profiles of 3 ERGs in the training set **(A)**, testing set **(D)**, entire set **(G)** and GEO set **(J)**. Kapan-Meier survival curves analyses and ROC analysis in predicting prognoses in the training set **(B,C)**, testing set **(E,F)**, entire set **(H,I)** and GEO set **(K,L)**.

To verify the prognostic potential of ERGs, 30 differentially expressed ERGs were evaluated for prognostic potential and ultimately, 6 ERGs were identified to be correlated with prognosis in the training set (Table 1). To prevent overfitting the prognostic signature, Lasso regression was applied and tenfold cross-validation for penalty parameter selection is shown in Supplementary Figure S2. Afterward, 3 ERGs were identified as key prognostic genes and in turn, the risk score of each patient was calculated based on the coefficient of these 3 prognostic ERGs: Risk score = 1.0132 * CCKBR + 0.2416 * HOXC6 + 0.7618 * POU4F1. The median risk score was used as a criterion for defining high-risk and low-risk groups. According to the OS of COAD patients, the ranked dot plot illustrated the survival status and expression of 3 key ERGs was showed in heatmap (Figure 2A). The OS of patients in the low-risk group was much better than those in the high-risk group by the Kaplan–Meier survival analysis (Figure 2B). The ROC curves demonstrated sensitivity and specificity of our signature in predicting OS (Figure 2C).

**TABLE 1:** Univariate COX regression analysis of 6 exosome-related genes in training set.

Genes	HR	Low 95%CI	Up 95%CI	*p* value
CCKBR	4.5298	2.2377	9.1699	<0.0001
CYP11A1	5.8097	1.5848	21.2979	0.0079
HOXC6	1.2669	1.0626	1.5104	0.0084
NEUROD1	1.6917	1.0611	2.6969	0.0272
UCHL1	1.4394	1.0049	2.0619	0.0470
POU4F1	3.0398	1.5067	6.1326	0.0019

### Validation of exosome-related prognostic signature

To certify the accuracy of risk scores, similar analysis was performed in the validation set including testing set, entire set, and GEO set. Based on same calculation formula, we calculated the risk score of each patient, determined the median risk score as the standard, and divided the patients into low-risk and high-risk group in the validation set. The distribution of risk score, survival status and the heatmap of 3 key ERGs were exhibited in the validation set (Figures 2D,G,J). Meanwhile, survival curves revealed that survival duration of the low-risk group was much longer in the validation set (Figures 2E,H,K). In addition, the AUC value of validation set confirmed the consistency and accuracy of this prognostic model (Figures 2F,I,L). The PCA analysis showed that patients in different risk groups tended to be well distinguished based on this classification (Supplementary Figure S3). We further validated the expression of 3 ERGs in colorectal cancer cell line HCT116, HT-29, Caco-2 by qRT-PCR. As shown in Supplementary Figure S4, the expression of CCKBR, HOXC6, and POU4F1 were significantly higher in colorectal cancer cell lines compared to those in FHC cells.

### Construction of predictive nomogram based on exosome-related prognostic signature

**FIGURE 3:**
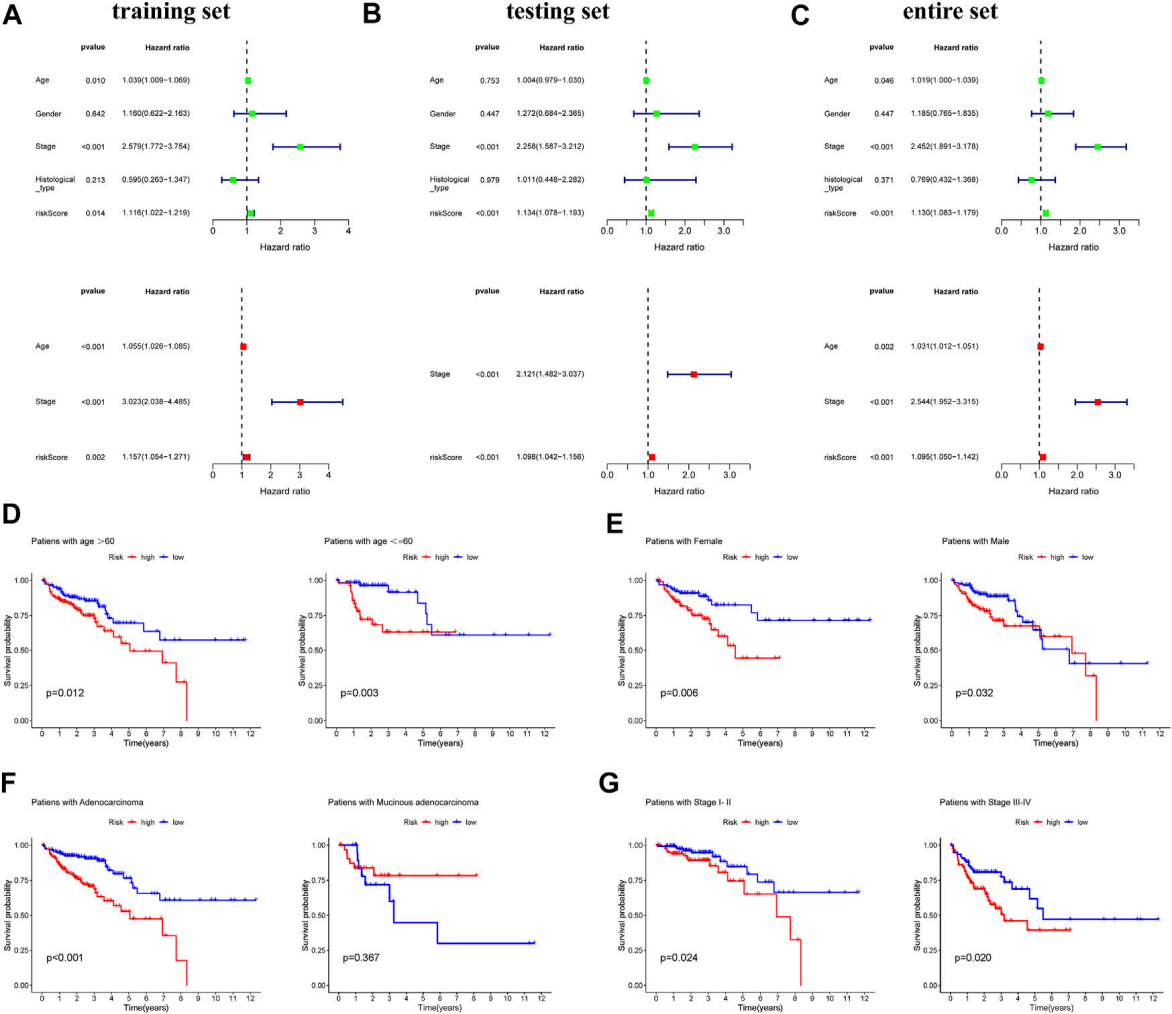
Analyses of the independent predictor of COAD patients. Univariate and multivariate COX regression analysis of clinical factors (age, gender, stage, histological types, and risk score) in the training set **(A)**, testing set **(B)**, and entire set **(C)**. Kapan-Meier survival curves reveal prognostic value of risk score in COAD patients with different ages **(D)**, genders **(E)**, histological types **(F)**, and stages **(G)**.

To assess whether risk score was independent prognostic factors, univariable and multivariable Cox regression analysis were performed in three set. The results showed that age, stage and risk score were independent prognostic factors and there was no significant specificity in gender and histological type (Figures 3A–C). We performed subgroup survival analysis to reveal the prognostic value of risk score in COAD patients who varied in age, gender, histological type and stage. The results revealed that the risk score could distinguish the prognosis in patients aging below and over 60 years old (Figure 3D), male and female patients (Figure 3E), patients with adenocarcinoma (Figure 3F), patients with different stages (Figure 3G).

**FIGURE 4:**
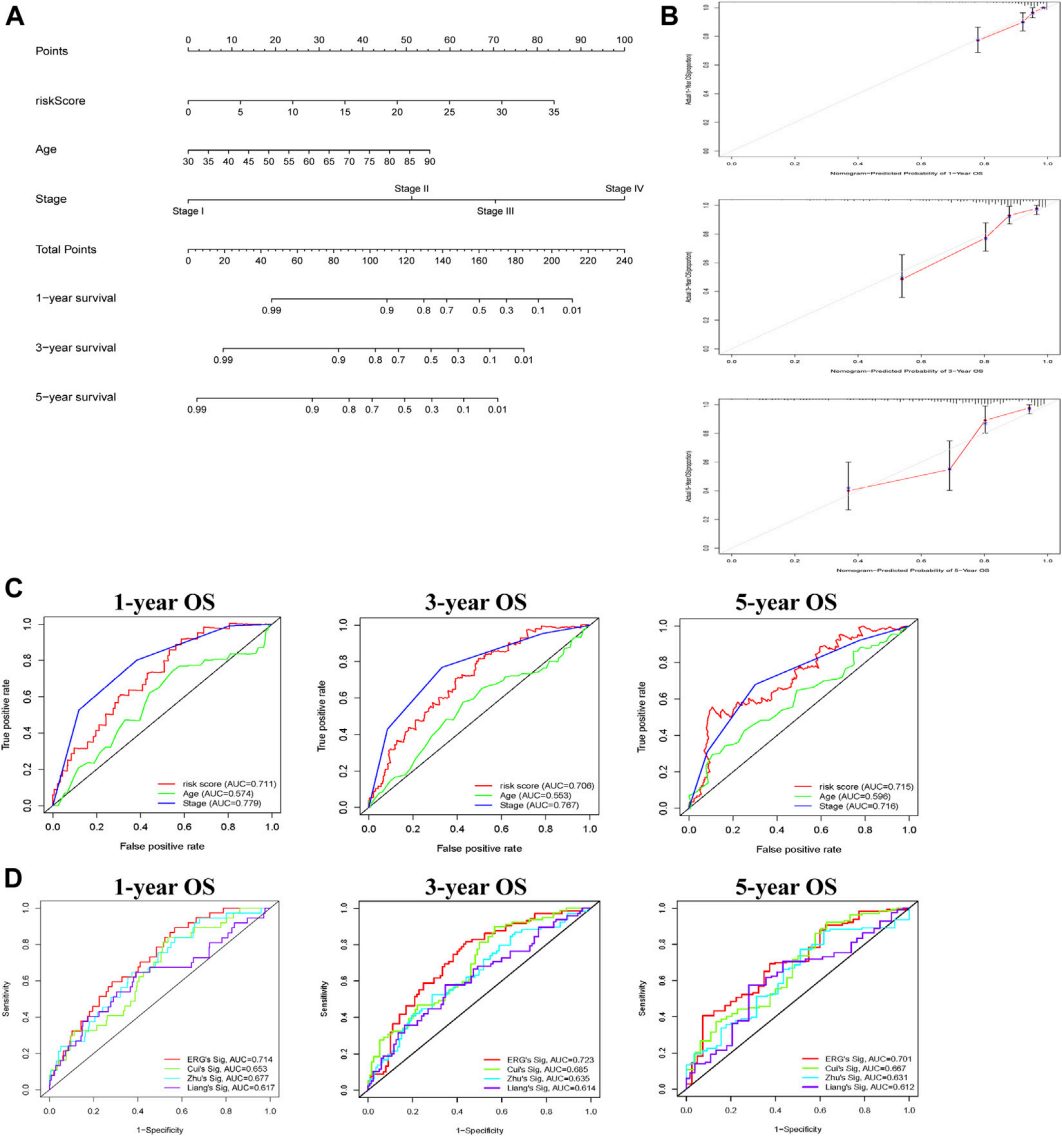
Construction of nomogram for predicting OS of patients with COAD. **(A)** The nomogram combining signature with clinicopathological features (risk score, age, stage) predicts probability of 1-, 3-, and 5-years OS. **(B)** The calibration plots reveal nomogram-predicted survival probabilities corresponded closely to the actually observed percentages. **(C)** The area under the curve (AUC) value of risk score and clinical factors was determined in 1-, 3- and 5-years OS. **(D)** The AUC of 3 ERGs-related signature and existing gene-related signature in 1-, 3-, and 5-years OS.

Subsequently, to predict OS, a prognostic nomogram was established based on the clinical factors and risk scores (Figure 4A). The calibration curves suggested that the survival rate as revealed by the nomogram was highly consistent with the actual survival rates, which indicated that the nomogram boasted important clinical significance (Figure 4B). Compared with the AUC value of clinical features, risk score showed good sensitivity and specificity in predicting OS of COAD patients (Figure 4C). To further prove the predictive role of this signature, we verified the performance differences between our signature and other recently reported gene signatures of prognostic model in COAD (Liang et al., 2020; Cui et al., 2021; Zhu et al., 2021). Based on the same patient set, the AUC of our TCGA signature for 1, 3, 5 years OS were significantly higher than exiting gene-related signature in COAD (Figure 4D).

### Immune status analysis

**FIGURE 5:**
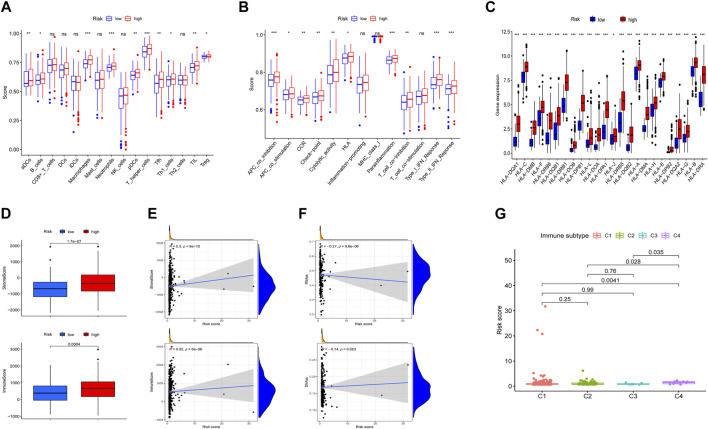
Situation of immune cell infiltration in high-risk and low-risk groups. **(A,B)** Comparison of the 16 tumors immune related cells and 13 immune-related functions. **(C)** The levels of HLA-related gene expression. **(D)** Comparison of ESTIMATES score, Immune score, and Stromal score. **(E,F)** Association between risk score and ESTIMATES score, Immune score, Stromal score, RNAss, and DNAss. **(G)** Comparison of the risk score in four immune infiltration subtypes. *p < 0.05, **p < 0.01, *** *p*< 0.001. ns, not significant.

To determine the biological processes involved, GSEA analysis was performed to analyze transcript information from high-risk and low-risk patients and identify representative KEGG pathways (Supplementary Figure S5). To better explain the relationship between immune status and risk score, tumor immune-related cells were compared in different risk groups and the ssGSEA results showed that multiple immune cells were elevated, so there was more immune activity in the high-risk group (Figure 5A). Next, 13 immune-related functions were also compared and the confirming the variability between the low-risk and high-risk groups (Figure 5B). Accordingly, we investigated the expression of human leucocyte antigen (HLA) related genes and found that the most HLA genes expression were higher in the high-risk group (Figure 5C). Moreover, we implemented the ESTIMATE algorithm and identified that stromal score and immune score were significantly higher in the high-risk group and were positively associated with the risk score (Figures 5D,E). RNA stemness score (RNAss) and DNA stemness score (DNAss) were employed to measure tumor stemness and both of them were significantly negatively associated with the risk score (Figure 5F). Different types of immune infiltration correspond to promotion and inhibition of tumor (Tamborero et al., 2018), such as C4 (lymphocyte depleted) which was significantly related with risk score (Figure 5G).

**FIGURE 6:**
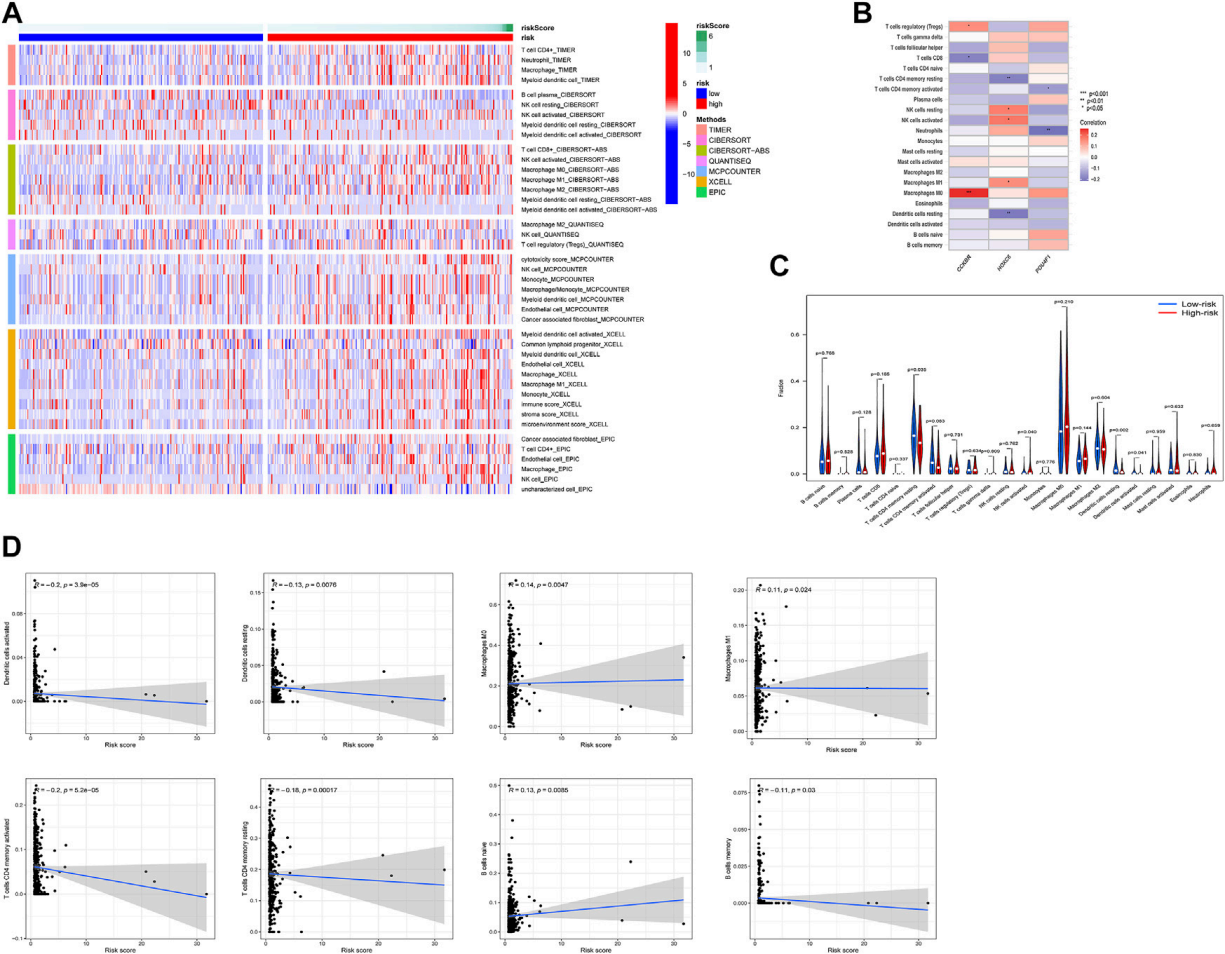
Relationship between risk score and tumor-infiltration immune cells in high-risk and low-risk groups. **(A)** The infiltration of TIICs was assessed by multiple databases (TIMER, CIBERSORT, CIBERSORT-ABS, QUANTISEQ, MCPCOUNTER, XCELL, and EPIC). **(B)** The relation between the expression of 3 ERGs and immune cell infiltration. **(C,D)** Comparison of TIICs and the association between risk score and TIICs. *p < 0.05, **p < 0.01, ***p < 0.001.

To explore tumor-infiltrating immune cells (TIICs) in the microenvironment of tumors, we used multiple databases to estimate infiltration of 21 types of TIICs (Figure 6A). The significant association between TIICs and the expression level of 3 prognostic ERGs were shown in Figure 6B. In addition, the percentage of each TIICs was compared and the significant correlation between risk score and TIICs was exhibited (Figures 6C,D).

### Mutation profile and microsatellite instability in ERG-based risk score

**FIGURE 7:**
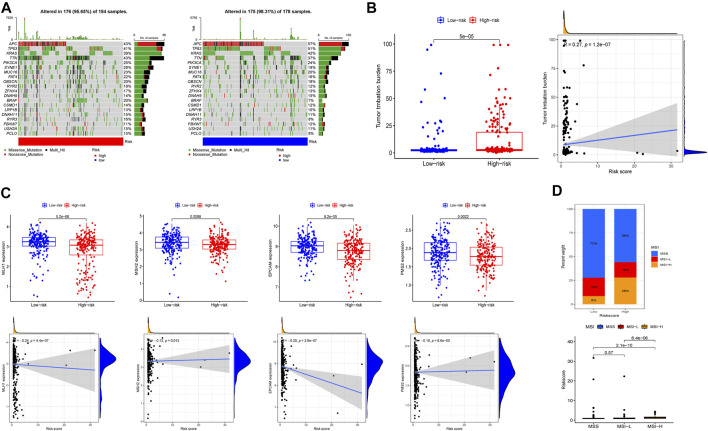
Tumor mutational burden, somatic mutation, and microsatellite instability (MSI) analysis in high-risk and low-risk groups. **(A)** The waterfall plot displayed the mutation of 20 genes. **(B)** The differences of TMB, the relationship between TMB and risk score. **(C)** The expression level of MLH1, MSH2, EPCAM, PMS2 and the relationship of expression and risk score. **(D)** The proportion of different microsatellite states and the risk score of different microsatellite states.

Tumor mutational burden is an important factor in tumor occurrence and development, so it can be used to predict the effectiveness of immune checkpoint inhibitors (ICIs) (Ai et al., 2020). In this study, cancer-related gene mutation data were used to assess the TMB level in two risk groups. The results showed that the top 3 mutation frequencies were APC, TP53, KRAS and the mutation frequencies of APC, TP53, and KRAS were lower in the high-risk group (Figure 7A). In addition, patients with high-risk score tended to have a higher TMB and TMB was positively correlated with risk score (Figure 7B). DNA mismatch repair (MMR) proteins is involved in the correction of mismatched bases in the process of DNA replication, and its inactivation will cause microsatellite instability (MSI) (Kim et al., 2022). We compared the expression level of MMR-related genes including MLH1, MSH2, PMS2, and MSH6 in different risk group and found that MMR-related genes expression was upregulated in low-risk group and negatively correlated with risk score (Figure 7C). Moreover, 28% of patients with MSI-high (MSI-H) were in high-risk group, which had larger number of patients than those with MSI-H in the low-risk group. Similarly, patients with MSI-H had higher risk score than patients in MSI-low (MSI-L) group and microsatellite stability (MSS) group (Figure 7D).

### Exosome-related prognosis signature and immune checkpoint

**FIGURE 8:**
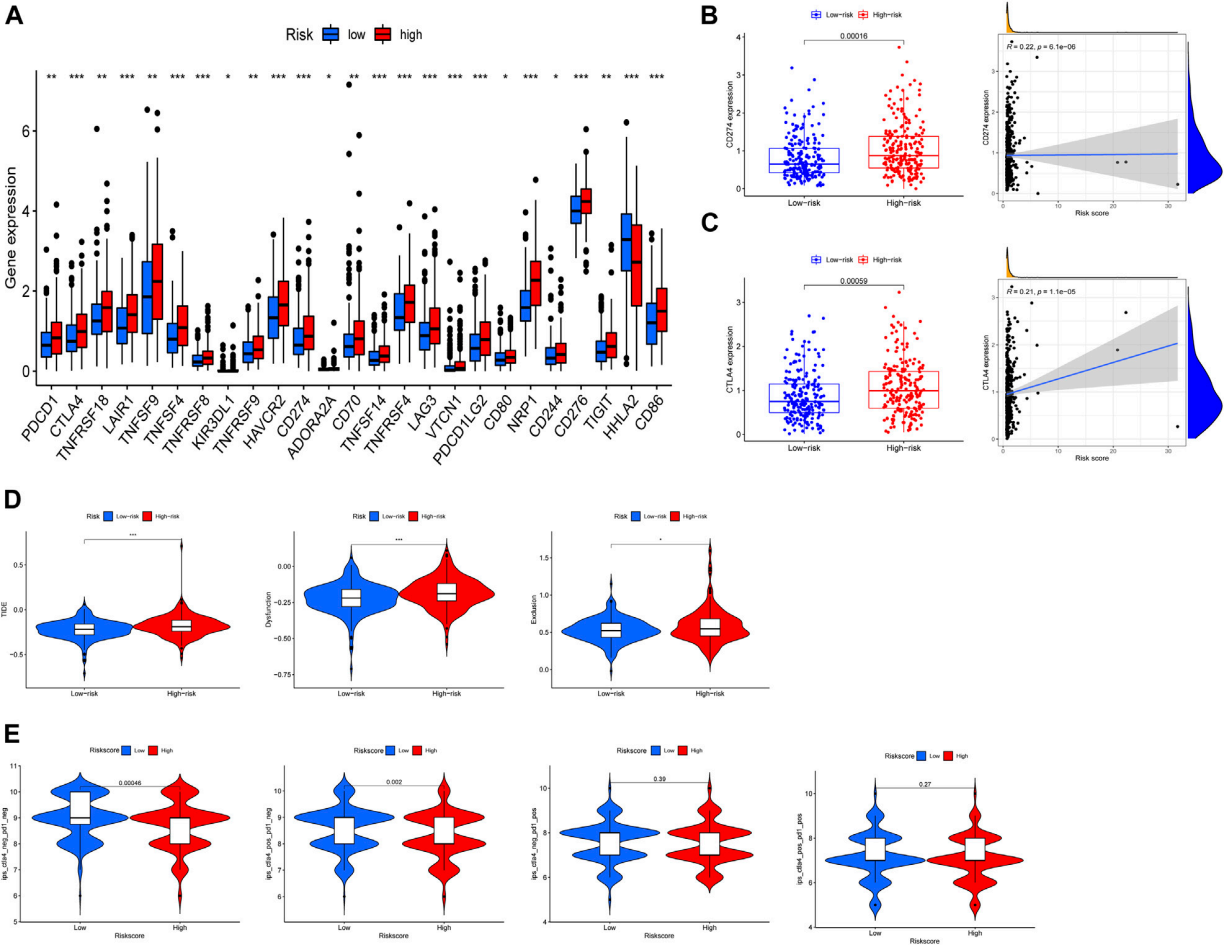
Immune checkpoint analysis in high-risk and low-risk groups. **(A)** The expression of immune checkpoints genes. **(B,C)** The relation between CD274, CTLA4 expression and risk score. **(D)** The differences of Tumor Immune Dysfunction and Exclusion (TIDE) scores. **(E)** The differences of IPS in patients with different risk group. *p < 0.05, **p < 0.01, ***p < 0.001.

Immune checkpoints help assess the patient’s immune response to immunotherapy (Yasunaga, 2020). The results demonstrated that 25 immune checkpoints expressed differently, and 24 of the 25 immune checkpoints had lower expression in the low-risk group, except for the HHLA2 gene, which had higher expression in the low-risk group (Figure 8A). In view of the distribution of immune checkpoint expression, we focused on CTLA4 and CD274 (PD-L1), and the results revealed that there was positively correlated of risk score and CTLA4 and PD-L1 expression (Figures 8B,C). To reveal the difference of immune evasion, we calculated the TIDE score of the two risk groups. The results showed that the TIDE scores in the high-risk group were significantly higher than that in low-risk group, which indicated that the patients in the high-risk group had greater potential for immune escape and worse effect on immunotherapy (Figure 8D). Furthermore, IPS analysis revealed the potential effects of anti-CTLA4 and anti PD-L1 between high-risk and low-risk group (Figure 8E).

### Exosome-related prognosis signature and chemotherapy sensitivity

**FIGURE 9:**
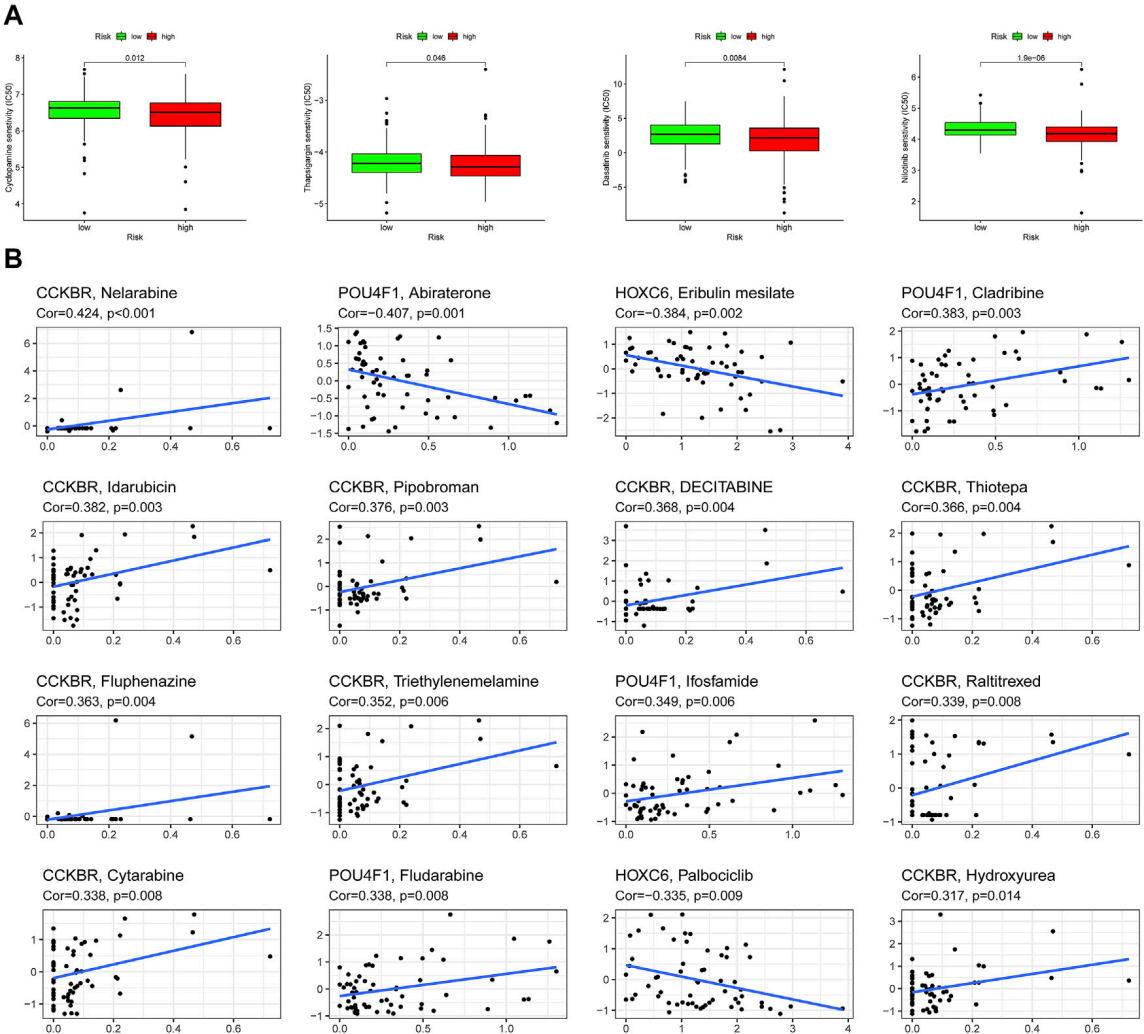
Chemosensitivity analysis in high-risk and low-risk groups. **(A)** Estimation of IC50 value for cyclopamine, thapsigargin, dasatinib, and nilotinib. **(B)** Scatter plot of the association between 3 ERGs expression and drug sensitivity.

Previous studies revealed that exosomes had a strong impact on drug resistance and induced drug resistance through a variety of mechanisms (Zhang and Yu, 2019; Namee and O’Driscoll, 2018). Considering that IC50 values reflect the drug sensitivity of cells to chemotherapeutic agents, four chemical drugs including cyclopamine, thapsigargin, dasatinib, nilotinib had been estimated. Patients in high-risk group had greater advantage in four chemotherapeutic drug sensitivity (Figure 9A). In addition, the relationship between expression of 3 prognostic ERGs and chemotherapy sensitivity was analyzed. The results indicated that 3 prognostic ERGs expression were strongly correlated with the sensitivity of some chemotherapeutic drug (Figure 9B). For instance, the higher expression of CCKBR was associated with upregulated sensitivity of tumor cells to nelarabine, idarubicin, pipobroman, decitabine, thiotepa, fluphenazine, triethylenemelamine, raltitrexed, cytarabine and hydroxyurea. Moreover, increased expression of POU4F1 was related to resistance to abiraterone and sensitivity to cladribine, while upregulated HOXC6 expression was associated with resistance to eribulin mesilate and palbociclib.

## Discussions

At present, COAD is one of the highest mortality cancers in the world, which still brings a heavy burden to families and society. Although there are many treatment methods, the curative effect is limited (Dekker et al., 2019). So far, exosomes have been widely involved in stem cells, immunity, microRNA, targeted drug delivery, cancer diagnosis and treatment. Exosomes have the characteristics of both promoting and inhibiting cancer. It has great potential in the field of tumor immunotherapy, thus, exosomes are expected to become immunotherapeutic targets and biomarkers for diagnosis, prognosis and treatment of COAD (Xu et al., 2020). Therefore, we established an exosome-related signature model and the results showed that this signature was an independent prognostic factor for COAD patients, and had the ability to identify tumor microenvironment, immune response and chemotherapy sensitivity in patients with different risk groups. Therefore, this model can be used to evaluate the prognosis of patients and provide insight for the treatment strategies in COAD patients.

Firstly, we collected data of COAD patients from the TCGA database and found that 30 ERGs were differentially expressed in tumor and normal samples. Then, 3 prognostic ERGs (CCKBR, HOXC6, and POU4F1) were identified to establish the prognostic signature and in this signature, patients were divided into high-risk and low-risk groups. The nomogram provided the predictive score for patients to confirm the survival possibility and the predictive ability further demonstrated by the ROC analysis. Moreover, the differences of tumor immune microenvironment, immune cell infiltration, immune checkpoint and sensitivity to drugs in two risk groups were also analyzed.

Cholecystokinin B receptor (CCKBR), also known as gastrin receptor, belongs to the G-protein coupled receptors (GPCRs). The activation of CCKBR stimulates the release of extracellular vesicles (EVs) (Conrad et al., 2020). Moreover, CCKBR are widely expressed in colorectal polyps, the activation of CCKBR occurs in the early stage of adenoma progression to cancer and promotes tumor progression (Smith and Watson, 2000). The occurrence of most colorectal cancer is directly correlated to the abnormal gastrin expression (Mao et al., 2014). Based on the aberrant expression of CCKBR and the functional binding interaction with gastrin, CCKBR might be a promising target for immunotoxin therapy (Grzmil et al., 2020; Li et al., 2022). Homeobox C6 (HOXC6) is a transcription factor that is implicated in the malignant progression of several cancers, including gastric cancer (Liu et al., 2020a; Lin et al., 2020), hepatocellular carcinoma (Li et al., 2018), colon carcinoma (Liu et al., 2020b). Furthermore, HOXC6 plays a key role in the regulation of multiple cellular signaling pathways during tumorigenesis, so it is considered as a novel biomarker for various cancers (Hewitson et al., 2004; Jeong et al., 2021). HOXC6 expression is also closely related to antitumor drug sensitivity (Kim et al., 2013; He et al., 2021). Our results suggested that upregulated HOXC6 expression was associated with resistance to chemotherapeutic drugs including eribulin mesilate and palbociclib. POU class 4 homeobox 1 (POU4F1), a stem cell-associated transcription factor, is considered to have tumor genetic function and promote tumor growth. Recently, it has been reported that POU4F1 enhance the proliferation and drug resistance of melanoma (Liu et al., 2020c). In addition, targeting POU4F1 may be a new effective treatment for trastuzumab (TRA) resistance in human HER2 positive breast cancer (Wu et al., 2020). Similarly, we found that the expression level of POU4F1 were significantly correlated with certain chemotherapy drugs.

Cancer stem cells (CSCs) are self-renewal cells found in tumors as normal stem cells (Kreso and Dick, 2014). CSCs can not only transform into cancer cells and enhance their resistance to chemoradiotherapy, but also secrete immunosuppressive cytokines such as transforming growth factor-β (TGF-β), interleukin-6 (IL-6), IL-10, and IL-13, which can induce immune evasion of tumor cells (Schatton and Frank, 2009; Zhang et al., 2019; Jahanafrooz et al., 2020). In addition, for many tumors, stemness indices are negatively correlated with the expression level of PD-L1 (Malta et al., 2018). In this study, risk score was negatively associated with stemness indices. Our results also found that the IC50 of cyclopamine, thapsigargin, dasatinib, nilotinib was lower in the high-risk group, indicating that the high-risk group with a lower stemness indices was more sensitive to chemotherapy drugs. Furthermore, PD-L1 expression was down-regulated in low-risk group with higher stemness indices, which was consistent with previous findings. However, the high-risk group with lower stemness indices shows stronger immune evasion ability, which may be related to the complex mechanism of immune evasion, involving the co-regulation of genes, metabolism, immune cells, blood vessels and other aspects. Metastatic CRC patients with mismatch repair deficient (dMMR) or microsatellite instability-high (MSI-H) respond better to immunotherapy (Andre et al., 2021). TMB refers to the total number of replacement, insertion and deletion mutations per megabyte in the exon coding region of evaluated genes in the genome of tumor cells. Highly mutated tumors are thought to contain an increased neoantigen load, making them immunogenic and responsive to immunotherapy (McGranahan et al., 2016). Large amounts of neoantigen production are associated with an enhanced response to checkpoint blocking and are thought to be the mechanism by which immune checkpoint inhibitors treat tumor tissues (Ott et al., 2017). Therefore, TMB can also effectively predict the efficacy of immunotherapy (Díaz-Gay and Alexandrov, 2021). Results of our study found that risk score was helpful to distinguish the COAD patients with different level of TMB, MMR, and MSI. Combined with the results of immune escape and immunotherapy analysis in different risk groups, suggesting that risk score is an effective indicator to reveal the effect of immunotherapy.

As an important regulator of tumor progression, the heterogeneity of tumor microenvironment can significantly affect the prognosis and treatment response of patients (Whiteside, 2008). The existence of tumor infiltrating lymphocytes (TILs) is related to the improvement of survival rate in COAD (Chandra et al., 2021). To identify the immune mechanism of this signature, we compared the difference of Tumor-infiltrating immune cells (TIICs) and its correlation with risk score was also revealed. We found that T-cells CD4 memory resting had a larger proportion in the low-risk group, while NK cells activated had a larger percentage in the high-risk group. High CD4 + T-cell density is associated with improved relapse free survival and disease-specific survival (Kuwahara et al., 2019). Tumor-derived exosomes induce proinflammatory cytokine expression and PD-L1 regulation in M0 macrophages through IL-6/STAT3 and TLR4 signaling pathways (Pucci et al., 2021). PD-L1 is highly expressed on tumor-associated macrophages and thus inhibits antitumor immune responses (Lin et al., 2018; Petty et al., 2021). Tumor cells can secrete exosomes carrying PD-L1, inhibit the activation of CD8+T-cells in tumor microenvironment or peripheral circulation, inhibit the proliferation of CD4+T-cells, upregulate the immunosuppressive function of Treg cells, downregulate the expression level of NKG2D in NK cells to inhibit immune killing, and finally promote immune evasion (Chen et al., 2018). Our results revealed that resting CD4 memory T-cells, resting dendritic cells and activated dendritic cells had higher levels in patients with lower risk score, while the level of activated NK cells was higher in high-risk group patients. In addition, risk score was positively correlated with M0 macrophages, M1 macrophages and naïve B cells, while had negative association with activated dendritic cells, resting dendritic cells, active CD4 memory T-cells, resting CD4 memory T-cells and memory B cells. Moreover, there were certain correlations between 3 ERGs and TIICs, including regulatory T cells (Tregs). These results revealed that the exosome-related signature constructed by ERGs can distinguish the different factors of tumor immune cells in COAD.

Compared with dendritic cells from healthy persons, the antigen presentation capacity of dendritic cells in COAD patients was impaired and the expression of costimulatory molecules was reduced (Orsini et al., 2013). Up-regulation of HLA-B/C may be beneficial to COAD patients (Michelakos et al., 2022), and this risk model help distinguish the different expression level of HLA. Furthermore, there were statistical differences between risk score and TIICs as well as four immune subtypes, suggesting that therapeutic potential of immunotherapy for COAD patients.

The presence of PD-L1 on the surface of tumor-derived exosomes is critical to the low response to immune checkpoint inhibitors (Poggio et al., 2019). In this study, we found that the expression of immune checkpoints such as CTLA4, PD-L1, and HAVCR2 was significantly positively correlated with risk score, thus patients with higher risk score may benefit more from immunotherapy. In addition to immunotherapy, the curative effect of chemotherapeutic drugs including cyclopamine, thapsigargin, dasatinib, nilotinib in different risk group was also assessed. As shown in the above results, patients in high-risk group were more sensitive to chemotherapeutic drugs. Therefore, this signature was constructed based on ERGs, which may be helpful to identify COAD patients with different risk score and provide beneficial treatment strategies.

It must be admitted that this study is not without inevitable limitations. First is the limited access to the public data set. Due to limited volume of data, the clinicopathological parameters analyzed in this study are not comprehensive, giving rise to errors or deviations. Second, we did not consider the heterogeneity of tumor microenvironment associated with exosomes. Finally, it is generally believed that prediction data without systematic validation lack the acceptability of clinical application. Therefore, the development of clinical application analysis will be the direction of our future work.

## Conclusion

In summary, we introduced a risk signature constructed by 3 ERGs for COAD patients and systematically evaluated its prognostic significance, and its role in tumor microenvironment and immune cell infiltration. What is more, the relationship of risk score and immune checkpoint, chemosensitivity was revealed, which may help to determine individual treatment strategies and give insights into advancing treatment methods.

## Data Availability

The data presented in the study are available from the Gene Expression Omnibus (GEO) database (https://www.ncbi.nlm.nih.gov/geo/) with the accession numbers GSE39582 and and The Cancer Genome Atlas (TCGA) database (https://portal.gdc.cancer.gov/) with the accession number TCGA COAD.
