# Supplementary material for: Development of an exosome-related and immune microenvironment prognostic signature in colon adenocarcinoma
Source: Front Genet. 2022 Sep 13;13:995644. doi: 10.3389/fgene.2022.995644 (PMC9513147; doi:10.3389/fgene.2022.995644)
Supplement: Supplementary file 1 [file Table1.DOCX]

**Table 1 Univariate COX regression analysis of 6 Exosome-related genes in training set.**

| Genes | HR | Low 95%CI | Up 95%CI | pvalue |
| --- | --- | --- | --- | --- |
| CCKBR | 4.5298 | 2.2377 | 9.1699 | <0.0001 |
| CYP11A1 | 5.8097 | 1.5848 | 21.2979 | 0.0079 |
| HOXC6 | 1.2669 | 1.0626 | 1.5104 | 0.0084 |
| NEUROD1 | 1.6917 | 1.0611 | 2.6969 | 0.0272 |
| UCHL1 | 1.4394 | 1.0049 | 2.0619 | 0.0470 |
| POU4F1 | 3.0398 | 1.5067 | 6.1326 | 0.0019 |
