# Supplementary figures and images for: Development of an exosome-related and immune microenvironment prognostic signature in colon adenocarcinoma
Source: Front Genet. 2022 Sep 13;13:995644. doi: 10.3389/fgene.2022.995644 (PMC9513147; doi:10.3389/fgene.2022.995644)

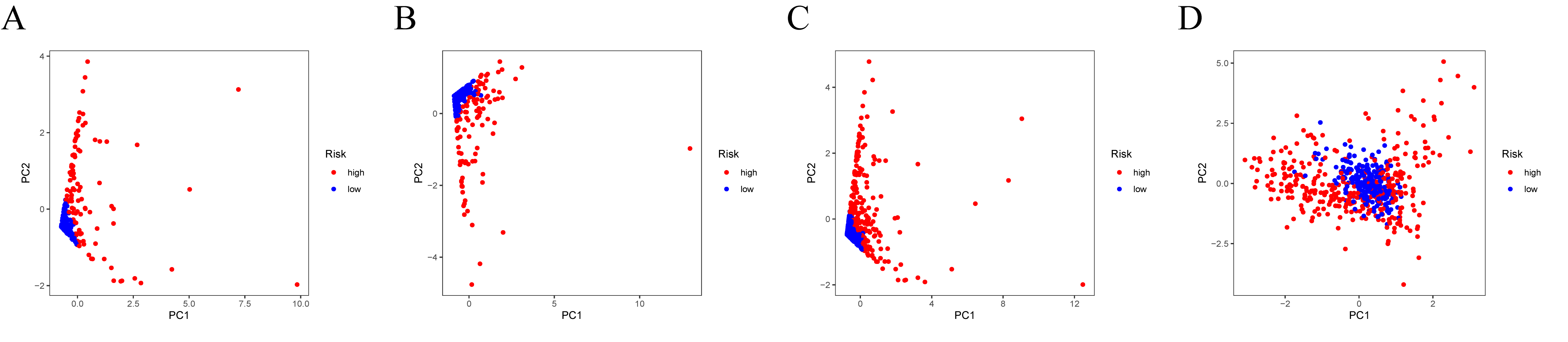

Supplement: Supplementary file 2 [file Image3.TIF]

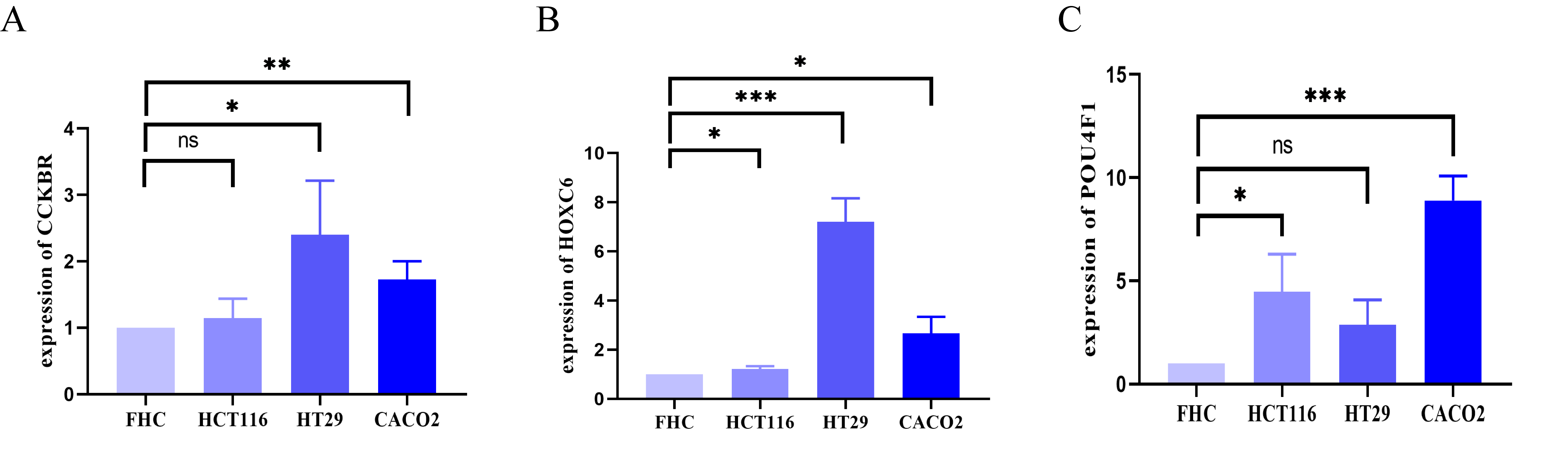

Supplement: Supplementary file 3 [file Image4.TIF]

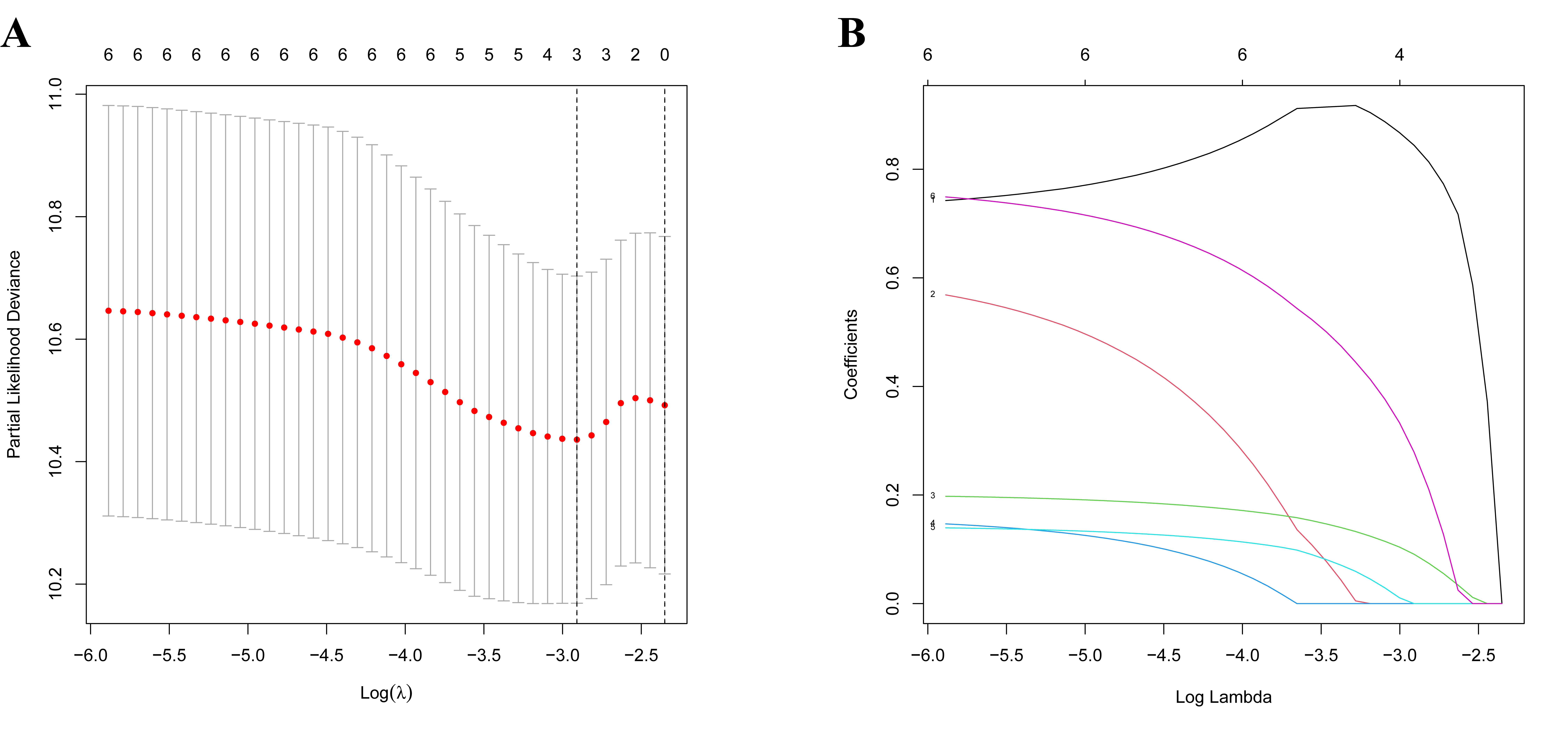

Supplement: Supplementary file 4 [file Image2.TIF]

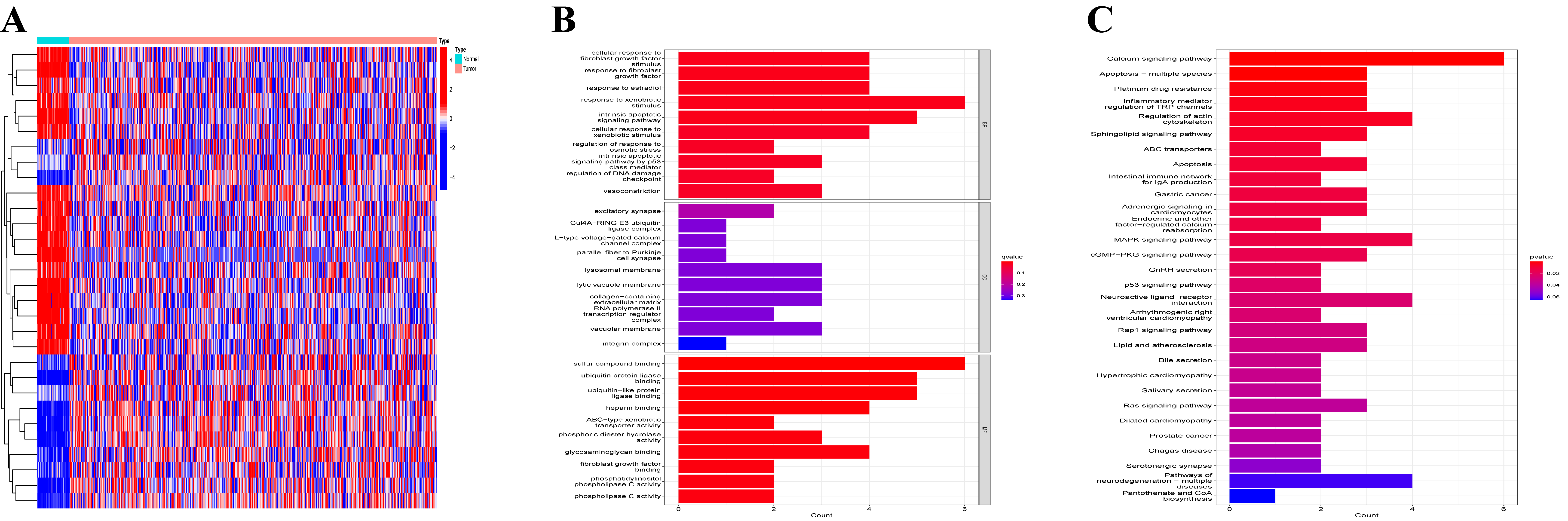

Supplement: Supplementary file 5 [file Image1.TIF]

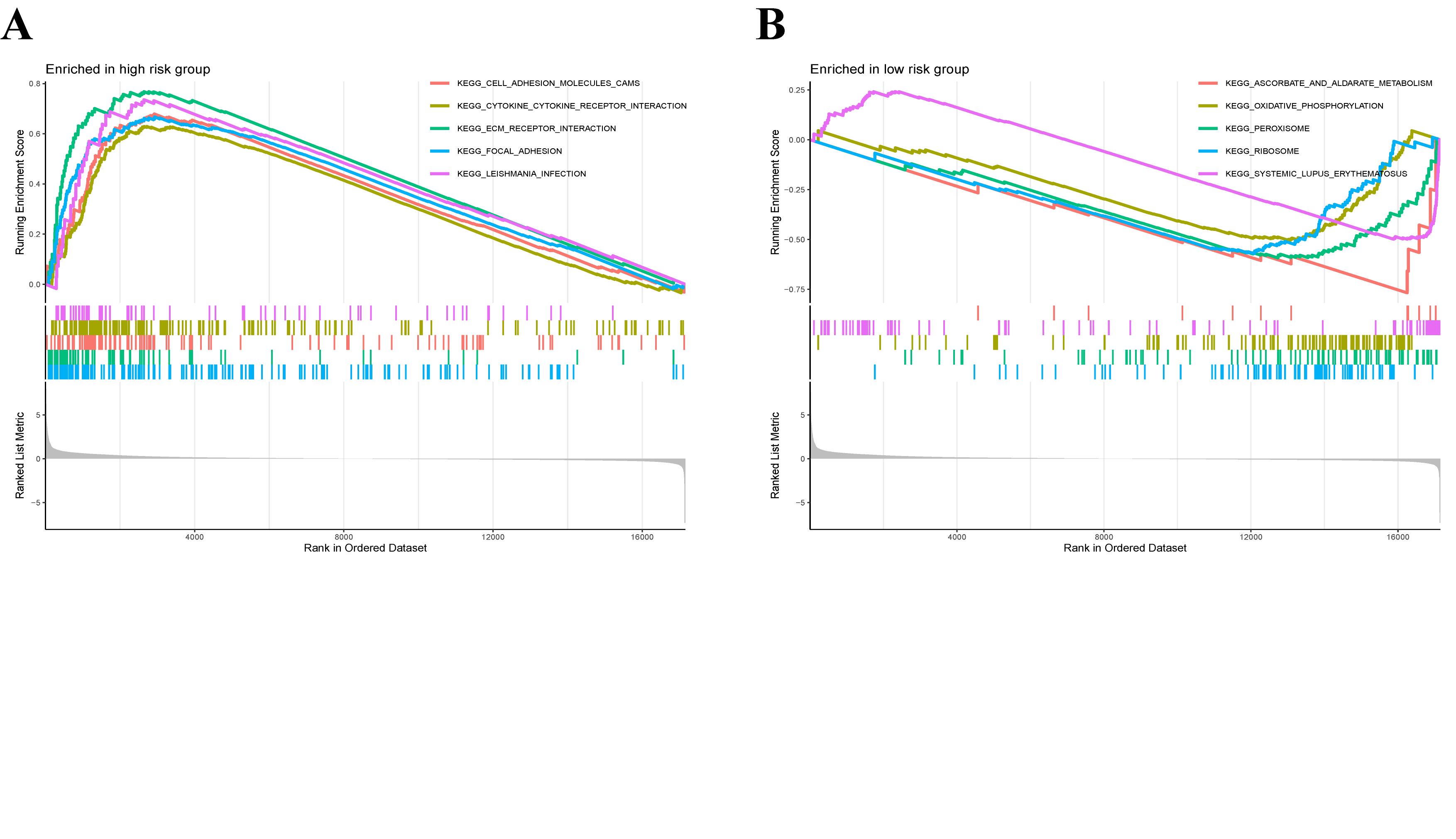

Supplement: Supplementary file 7 [file Image5.TIF]
