# Supplementary material for: Development of an exosome-related and immune microenvironment prognostic signature in colon adenocarcinoma
Source: Front Genet. 2022 Sep 13;13:995644. doi: 10.3389/fgene.2022.995644 (PMC9513147; doi:10.3389/fgene.2022.995644)
Supplement: Supplementary file 6 [file Table2.DOCX]

| Covariates | Type | Entire set | Testing set | Training set | Pvalue |
| --- | --- | --- | --- | --- | --- |
| Age | <=65 | 175(41.97%) | 87(41.83%) | 88(42.11%) | 1 |
|  | >65 | 242(58.03%) | 121(58.17%) | 121(57.89%) |  |
| Gender | FEMALE | 193(46.28%) | 101(48.56%) | 92(44.02%) | 0.4059 |
|  | MALE | 224(53.72%) | 107(51.44%) | 117(55.98%) |  |
| Stage | Stage I | 72(17.27%) | 36(17.31%) | 36(17.22%) | 0.7831 |
|  | Stage II | 160(38.37%) | 83(39.9%) | 77(36.84%) |  |
|  | Stage III | 117(28.06%) | 54(25.96%) | 63(30.14%) |  |
|  | Stage IV | 57(13.67%) | 30(14.42%) | 27(12.92%) |  |
|  | unknow | 11(2.64%) | 5(2.4%) | 6(2.87%) |  |
| T | T1 | 9(2.16%) | 6(2.88%) | 3(1.44%) | 0.7406 |
|  | T2 | 74(17.75%) | 36(17.31%) | 38(18.18%) |  |
|  | T3 | 284(68.11%) | 143(68.75%) | 141(67.46%) |  |
|  | T4 | 49(11.75%) | 23(11.06%) | 26(12.44%) |  |
|  | unknow | 1(0.24%) | 0(0%) | 1(0.48%) |  |
| M | M0 | 311(74.58%) | 161(77.4%) | 150(71.77%) | 1 |
|  | M1 | 57(13.67%) | 30(14.42%) | 27(12.92%) |  |
|  | unknow | 49(11.75%) | 17(8.17%) | 32(15.31%) |  |
| N | N0 | 247(59.23%) | 127(61.06%) | 120(57.42%) | 0.6541 |
|  | N1 | 98(23.5%) | 45(21.63%) | 53(25.36%) |  |
|  | N2 | 72(17.27%) | 36(17.31%) | 36(17.22%) |  |

**Table S1 Clinical features of COAD patients**

**Table S2 Primer sequence of genes in qRT-PCR**

|  | Forward sequence | Reverse sequence |
| --- | --- | --- |
| HOXC6 | ACAGACCTCAATCGCTCAGGA | AGGGGTAAATCTGGATACTGGC |
| CCKBR | GGGACACGAGAATTGGAGCTG | AACCGCCTTGCAGATGACG |
| POU4F1 | GGGCAAGAGCCATCCTTTCAA | CTGTTCATCGTGTGGTACGTG |
| β-actin | CATGTACGTTGCTATCCAGGC | CTCCTTAATGTCACGCACGAT |
